# A Case of Histoplasmosis Mimicking Primary Lung Cancer With Liver Metastasis

**DOI:** 10.7759/cureus.13517

**Published:** 2021-02-23

**Authors:** Emmanuel Fohle, Rishi Seth, Abhishek Matta

**Affiliations:** 1 Internal Medicine, University of North Dakota, Fargo, USA; 2 Internal Medicine, Sanford Health, University of North Dakota School of Medicine, Fargo, USA

**Keywords:** histoplasmosis, hairy cell leukemia, lung cancer

## Abstract

Histoplasmosis is usually self-limiting in healthy individuals but often fatal in immunocompromised patients. It can mimic primary lung malignancy and liver metastasis, causing a delay in appropriate therapy. We report a case of a 58-year-old male, with a 20 pack-year smoking habit, who presented with a three-week history of persistent fevers and productive cough with night sweats. Computed tomography (CT) scan of chest, abdomen and pelvis showed findings suggestive for primary lung malignancy associated with liver metastasis. Liver biopsy showed budding yeast. Bronchoalveolar lavage (BAL) fluid grew fungal organisms. Urine and serology were positive for histoplasmosis. Patient was pancytopenic, hence, we decided to evaluate further with a bone marrow biopsy which revealed underlying hairy cell leukemia. In the case of disseminated histoplasmosis, a high degree of suspicion towards any immunosuppressive condition should be entertained and any signs should be promptly investigated.

## Introduction

Histoplasmosis is one of the most endemic mycoses in the United States of America and is caused by *Histoplasma capsulatum*, a dimorphic fungus found in fertile moist soil contaminated by bird and bat droppings [1,2]. It is endemic in the central eastern United States, mostly in the Ohio and Mississippi River valleys [3]. *H. capsulatum* is acquired by inhalation of the mycelia fragments of the fungus. Clinically, it may manifest in three forms: acute pulmonary histoplasmosis, chronic pulmonary histoplasmosis, and disseminated histoplasmosis [4]. In most cases, acute histoplasmosis is asymptomatic and can manifest as self-limited pneumonia. However, in immunocompromised patients, such as in those with human immunodeficiency virus (HIV), it can cause clinically significant disease and is potentially fatal. Clinical manifestations of disseminated histoplasmosis include fevers, night sweats, weight loss, hepatosplenomegaly, cutaneous lesions and involvement of any other organ. In some cases, the manifestations can mimic primary or metastatic lesions leading to delay in appropriate treatment, especially in immunocompetent individuals [5]. We report a case of disseminated histoplasmosis in a patient with previously undiagnosed hairy cell leukemia who presented with persistent fevers and productive cough with radiological findings mimicking primary lung malignancy with liver metastasis, which prompted screening for immunosuppressive conditions.

## Case presentation

**Figure 1:**
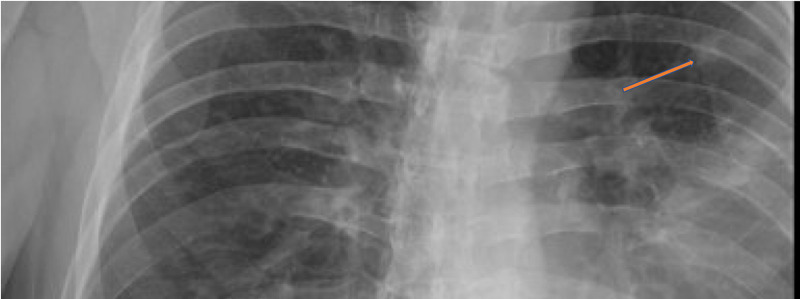
Chest x-ray with left nodular density

**Figure 2:**
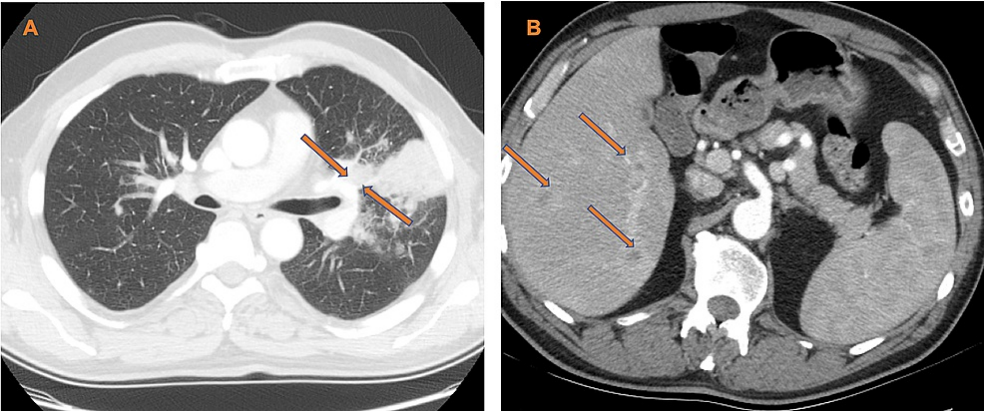
A, CT chest showing mass in left upper lobe with invasion of the left hilar structures; B, hepatic masses

A 58-year-old male, with 20 pack-year smoking history, initially presented to his primary care physician with concerns of persistent fevers, night sweats and productive cough with whitish phlegm. He was treated for presumed community-acquired pneumonia with azithromycin 500 mg for one day followed by 250 mg daily for four days. His symptoms did not improve. He had chest x-ray which showed left upper lobe infiltrate and 1 cm nodule-like density overlying the anterior third rib and a 5 cm nodular density between the fifth and sixth rib (Figure 1). CT chest revealed 7.7x6.5 cm mass in the left upper lobe concerning for primary malignancy (Figure 2A) with numerous ring-enhancing hypodense hepatic masses (Figure 2B). The patient was admitted to the hospital for further management.

On admission, his temperature was 37^o^C, heart rate 101 bpm, blood pressure 128/50 mmHg and respiratory rate 18 breaths/min saturating 99% on room air. On examination, the patient had a decreased breath sounds in the left lower lobe. He was empirically started on broad-spectrum antibiotics.

Initial laboratory findings are summarized below (Table 1). Given chest x-ray and CT scan findings, the patient underwent bronchoscopy which revealed patent airways. Bronchoalveolar lavage (BAL) cytology was positive for a fungal organism but negative for malignancy. BAL culture grew *Candida glabrata,* but this could not explain the clinical presentation. The patient subsequently underwent ultrasound-guided liver biopsy which showed the presence of fungal organism as budding yeasts highlighted by Grocott's methenamine silver (GMS) stain. There was no evidence of carcinoma. Fungal antibodies were negative but both serum and urine histoplasma antigen were positive. The patient was started on amphotericin B liposome 400 mg daily which was later changed to itraconazole 200 mg twice daily due to worsening kidney function.

**Table 1:** Laboratory findings on the day of admission, day 7, 18 and 29 of hospitalization RBC: red blood cells, WBC: white blood cells, BUN: blood urea nitrogen, ALP: alkaline phosphatase, ALT: alanine transaminase, AST: aspartate aminotransferase

	Admission	Day #7	Day#18	Day#29	Reference range
Hemoglobin	11.1	7.9	7.8	8.4	13-15 g/dL
RBC	3.39	2.58	2.58	2.73	4.6-6.8 x 106/mcL
WBC	1.8	1.3	0.5	8.4	3.6-10.3 x 103/mcL
Platelet	233	189	122	166	140-420 x 103/mcL
Absolute neutrophils	1,300	770	400	783	/uL
Absolute lymphocytes	0.3	0.2	0.1	0	0.8-4.1 K/uL
Blood glucose	107	126	103	105	70-100 mg/dL
Sodium	134	136	139	138	135-145 mmol/L
Potassium	4.4	3.9	3.5	3.1	3.7-5.1 mmol/L
Chloride	100	107	107	110	96-110 mmol/L
Bicarbonate	24	33	23	20	22-32 mmol/L
BUN	19	16	23	9	6-24 mg/dL
Creatinine	0.83	1.96	0.89	0.76	0.6-1.3 mg/dL
Calcium	9.6	7.6	7.7	7.1	8.5-10.5 mg/dL
Bilirubin total	0.5	0.4	0.5	0.5	0.2-1.2 mg/dL
ALP	304	321	468	299	30-150 U/L
ALT	76	32	40	99	0-35 U/L
AST	55	34	34	58	0-35 U/L

**Figure 3:**
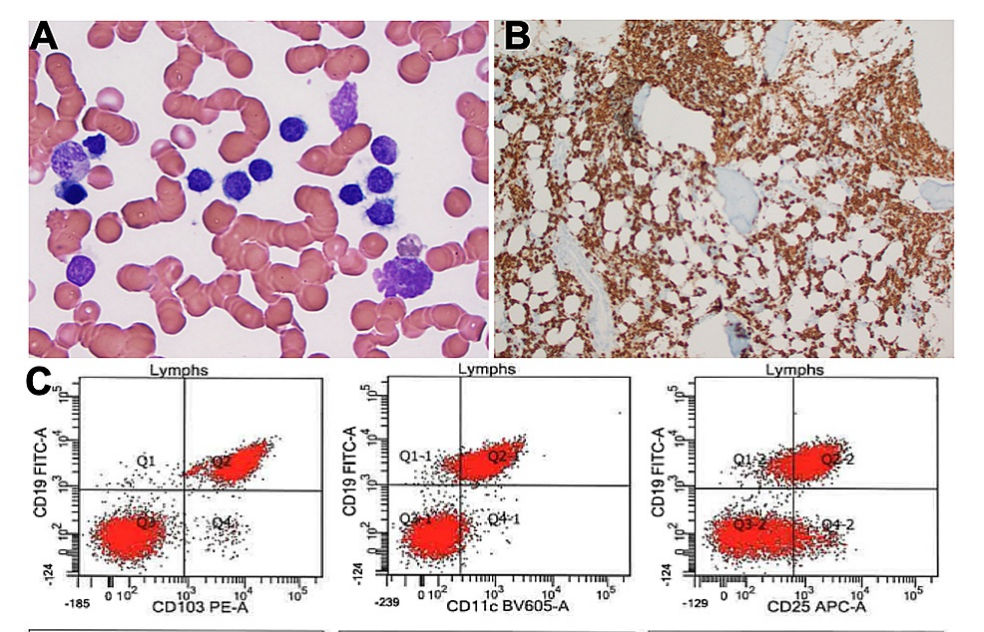
A, Bone marrow morphologic features (hematoxylin and eosin [H&E], x40). B, Bone marrow with immunostain for CD20 (x40). C, Selected immunophenotypes showing a clonal B-cell population positive for CD103, CD11c, CD25.

He continued to have persistent fevers and worsening leukopenia. HIV and hepatitis B serologies were negative. Given his systemic disseminated infection and suboptimal response to appropriate therapy, we decided to perform a bone marrow biopsy to rule out lymphoproliferative disorders and hemophagocytic lymphohistiocytosis. Bone marrow aspirate and biopsy revealed hairy cell leukemia involving a cellular 50% bone marrow (Figure 3A, 3B). Flow cytometry showed a monoclonal B lymphoid population, positive for CD19, CD11c, CD25, and CD103 (Figure 3C). Patient was started on cladribine and rituximab alongside levofloxacin, acyclovir and Bactrim for prophylaxis. Patient’s fever resolved and he was discharged home. During subsequent follow-up, patient was doing well, fevers resolved, and white blood cell count recovered (Table 2).

**Table 2:** Post hospitalization follow up laboratory findings RBC: red blood cells, WBC: white blood cells

	3 month follow up post treatment	6 month follow up post treatment	Reference range
Hemoglobin	14.5	13.1	13-15 g/dL
RBC	4.58	4.17	4.6-6.8 x 106/mcL
WBC	6.3	5.1	3.6-10.3 x 103/mcL
Platelet	149	154	140-420 x 103/mcL
Absolute neutrophils	4,500	4,200	/uL
Absolute lymphocytes	0.8	0.4	0.8-4.1 K/uL

## Discussion

Histoplasmosis is caused by the dimorphic fungu*s H. capsulatum *via inhalation of fungus in dust contaminated with bird or bat excrement. In the United States of America, it is especially endemic around the Ohio and Mississippi River valleys. In healthy individuals, it can present as a self-limited disease. However, in immunosuppressed individuals, it presents as a disseminated disease. As a result of impaired cellular immunity, the fungus will continue to reproduce and spread via lymphatic or hematogenous circulation [6]. Symptoms for disseminated histoplasmosis are often nonspecific, such as persistent fevers, night sweats, weight loss, coughs, cutaneous lesions, oral ulcers, hepatosplenomegaly, encephalitis and pericarditis [7]. Radiographic manifestations may mimic tuberculosis, sarcoidosis, malignancy and community-acquired pneumonia [8]. Laboratory findings include pancytopenia, elevated liver enzymes and occasionally adrenal insufficiency [9]. Testing strategy includes histoplasma serology, urine antigen and often bronchoalveolar lavage. Serology can be negative in the first month given it takes more than four weeks to mount an antibody response.

In our case, the patient presented with persistent fevers, night sweats and cough despite antibiotics. Given he is a smoker, the initial CT scan was highly suspicious for primary lung malignancy with metastasis to the liver. Another differential was post-obstructive pneumonia or another infectious cause in a setting of presumed malignancy causing immunosuppression. Following BAL, ultrasound-guided liver biopsy and fungal serology, he was diagnosed with disseminated histoplasmosis. Given his systemic disseminated infection and suboptimal response to appropriate therapy, we pursued bone marrow biopsy which showed hairy cell leukemia. There are few case reports about concurrent histoplasmosis and hairy cell leukemia. Clinically, features of disseminated histoplasmosis and hairy cell leukemia often overlap. They both present with fevers, weight loss, night sweats, hepatosplenomegaly, pancytopenia and pulmonary infiltrate [10].

This case demonstrates the need to have a high index of suspicion of immune suppressive conditions in individuals with disseminated histoplasmosis. It also highlights the difficulties in making the diagnosis of histoplasmosis especially in a patient from a non-endemic area with no clear evidence of exposure, immunodeficiency and similarity with lung malignancy on images. In addition, this case report is also relevant given low-dose CT is nationally used for the lung cancer screen. Research studies have shown promise for early-stage lung cancer detection using low- dose CT scan. Even though histoplasmosis is similar to lung malignancy on chest images, Starnes et al. found that screening with CT scan for lung cancer screen can be done effectively in an area endemic with histoplasmosis while minimizing benign biopsies [11]. Our patient was not from an endemic area, but we relied on our clinical suspicion not to pursue a lung biopsy.

## Conclusions

Histoplasmosis can present as self-limiting pneumonia but is often disseminated in immunocompromised individuals, sometimes leading to death. In the case of disseminated histoplasmosis in an otherwise healthy patient, an attempt should be made to identify an underlying cause of immunosuppression such as malignancy, AIDS or medications. The astute clinician should be aware that radiographic findings can mimic lung malignancy thereby causing a delay in appropriate therapy and an unnecessary lung biopsy.
